# Supplementary material for: Long-read, whole-genome sequencing and chemotherapy response of two patient-derived organoids from a TP53- and KRAS-mutant ovarian carcinoma
Source: bioRxiv. 2026 Jul 10:2026.07.06.736185. Preprint. [Version 1] doi: 10.64898/2026.07.06.736185 (PMC13371062; doi:10.64898/2026.07.06.736185)

bioRxiv preprint doi: https://doi.org/10.64898/2026.07.06.736185; this version posted July 10, 2026. The copyright holder for this preprint (which was not certified by peer review) is the author/funder, who has granted bioRxiv a license to display the preprint in perpetuity. It is made available under a CC-BY 4.0 International license.

Table 1. Single nucleotide variants reported in a clinical sequencing report from tumor sequencing at Tempus. Allele Fraction (AF) is reported from the clinical sequencing report (Clinical Genomics AF), as well as from long-read sequencing of OC104 and OC109 PDOs. “na” indicates that the variant was not detected/reported.

Supplemental Figure 1. Long-read sequencing metrics. (A) Mean coverage across the genome. (B) N50, or the read size for which the sum of it and all larger reads make up half of the total number of base pairs sequenced. (C) Total number of reads for each sample. For each figure panel, individual data is shown for each sample (PDO name labelled) and the mean is represented by the horizontal bar.

Supplemental Figure 2. IGV diagrams of LRS single-nucleotide polymorphism nonsynonymous variant calls from PDOs. (A) NCOR2 p.Arg1625Cys caused by a G>A nucleotide variant. (B) TSC2 p.Ala583Thr caused by a G>A nucleotide variant. (C) CTNNA2 p.Glu282Val caused by an A>T nucleotide variant. For each panel, reads are phased into haplotypes 1 and 2 or unassigned. Haplotypes are randomly assigned.

Supplemental Figure 3. Copy number analysis of PDOs. (A) Copy number across genome of PDOs, Top: OC104, Bottom, OC109. For each chromosome, segment copy number ratios are shown on the left side, and copy number ratio by bin on the right side. Genes with an absolute log2 fold change >2 are labelled. (B) Side-by-side copy number ratios of PDOs. (C) Chromosome 9p locus that contains the CDKN2A gene (yellow vertical bar). Gray points represent copy number bin values, and orange horizontal lines indicate the segment level copy number ratio.

bioRxiv preprint doi: https://doi.org/10.64898/2026.07.06.736185; this version posted July 10, 2026. The copyright holder for this preprint (which was not certified by peer review) is the author/funder, who has granted bioRxiv a license to display the preprint in perpetuity. It is made available under a CC-BY 4.0 International license.

Supplemental Figure 4. Distribution of lengths of insertions and deletions (Indels) by step in the filtering process. (A) OC104 insertions, (B) OC104 deletions, (C) OC109 insertions, and (D) OC109 deletions. Indel length is described in base pairs. CuteSV indicates indels called by the cute SV SV caller. Sniffles indicates indels called by the sniffles2 SV caller. Merged indicates the SVs merged by MAVIS, prior to filtering. MAVIS indicates the SVs after MAVIS's filtering step. GnomAD indicates those SVs that passed filters removing regions of very high or very low coverage in the population.

Supplemental Figure 5. IGV diagrams of LRS translocation calls from PDOs. (A) Breakpoint locations for BND1 between chr19 (left) and chrX (right). (B) Breakpoint locations for BND2 between chr17 (left) and chrX (right). (C) Breakpoint locations for BND3 between chr X (far left), chr21 (middle left), chr6 (middle right), and chrX (far right). (D) Breakpoint locations for BND4 between chr2 (left) and chr21 (right). Annotated genes and RepeatMasker repeat regions are shown below alignment tracks. For each panel, reads are phased into haplotypes 1 and 2 or unassigned.

Supplemental Figure 6. Signal tracks of differentially methylated promoter regions of (A) BRCA2, (B) HNF1A, (C) MDM4, (D) MSI2, and (E) SETBP1 showing 5-methyl cytosine methylation, the detected DMR, and the gene annotation track.

Supplemental Table 1. Organoid media formulations, suppliers, and catalog numbers.

Supplemental Table 2. Single nucleotide variants prioritized from long-read sequencing of OC104 and OC109 PDOs with database annotations. Each SNV is annotated with effect predictions (AlphaMissense, PolyPhen, SIFT) with the category with score in parentheses. The

bioRxiv preprint doi: https://doi.org/10.64898/2026.07.06.736185; this version posted July 10, 2026. The copyright holder for this preprint (which was not certified by peer review) is the author/funder, who has granted bioRxiv a license to display the preprint in perpetuity. It is made available under a CC-BY 4.0 International license.

total number of ovarian cancer patients in the AACR GENIE database with the exact variant are shown, as well as breakdown by subtype for either exact variant match or any alteration to the gene. Genes are also annotated for presence/frequency in COSMIC (occurrence in cancer) and GnomAD (frequency in population) databases. AF = allele fraction, TSG = tumor suppressor gene, OG = oncogene.

bioRxiv preprint doi: https://doi.org/10.64898/2026.07.06.736185; this version posted July 10, 2026. The copyright holder for this preprint (which was not certified by peer review) is the author/funder, who has granted bioRxiv a license to display the preprint in perpetuity. It is made available under a CC-BY 4.0 International license.

Supplemental Figure 1

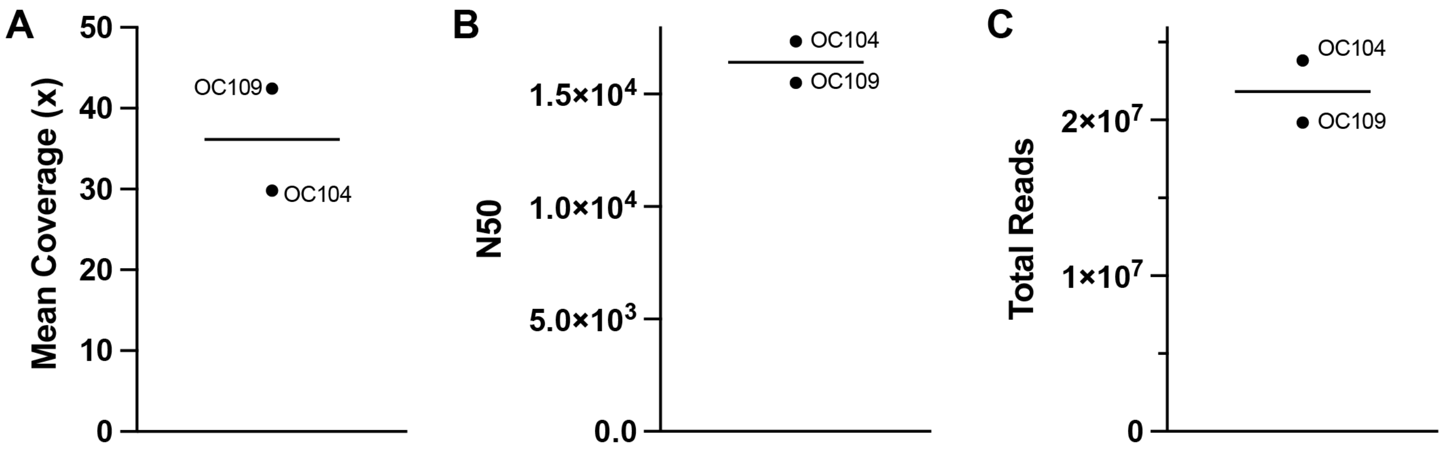

bioRxiv preprint doi: https://doi.org/10.64898/2026.07.06.736185; this version posted July 10, 2026. The copyright holder for this preprint (which was not certified by peer review) is the author/funder, who has granted bioRxiv a license to display the preprint in perpetuity. It is made available under a CC-BY 4.0 International license.

Supplemental Figure 2

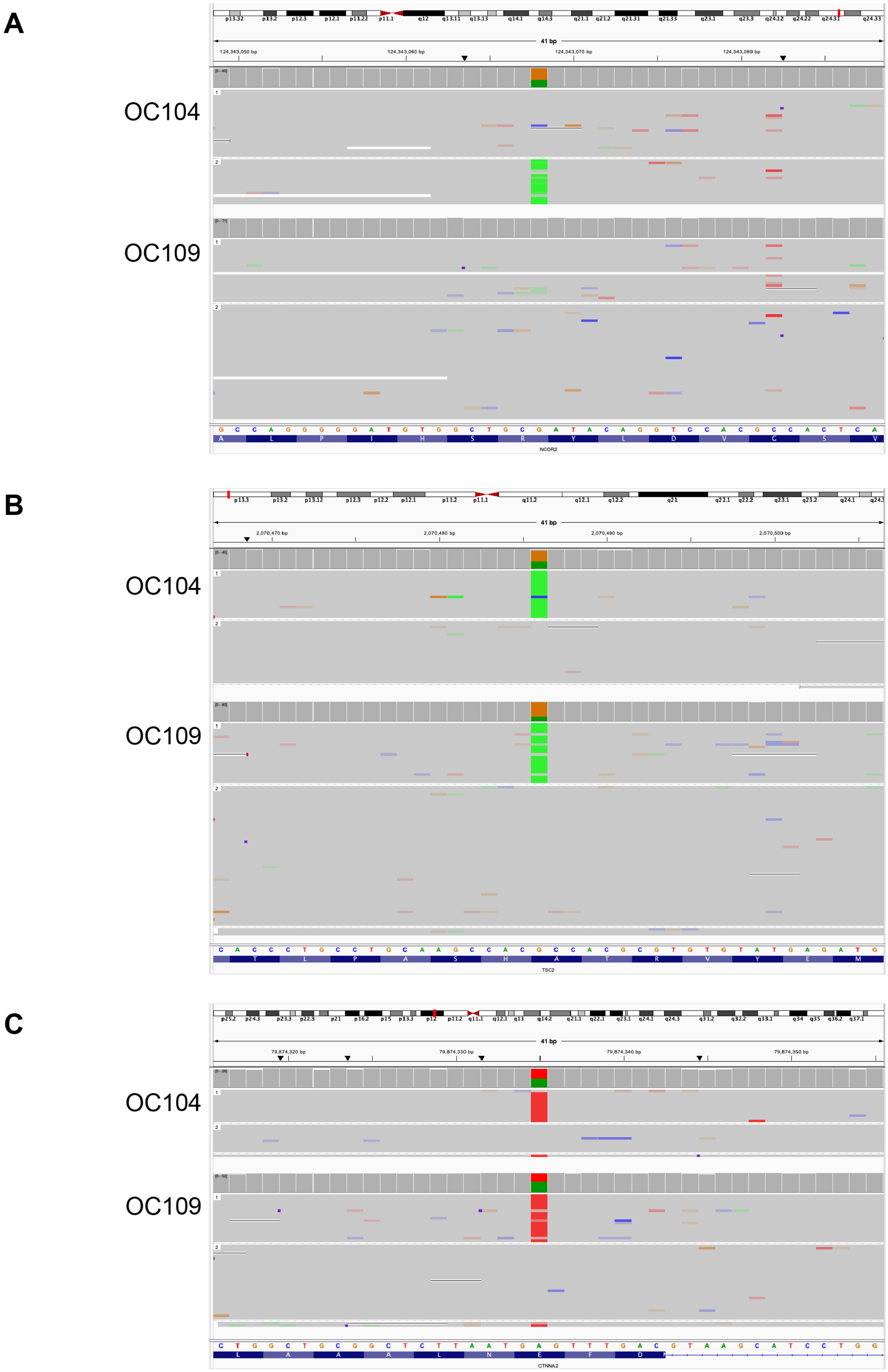

bioRxiv preprint doi: https://doi.org/10.64898/2026.07.06.736185; this version posted July 10, 2026. The copyright holder for this preprint (which was not certified by peer review) is the author/funder, who has granted bioRxiv a license to display the preprint in perpetuity. It is made available under a CC-BY 4.0 International license.

Supplemental Figure 3

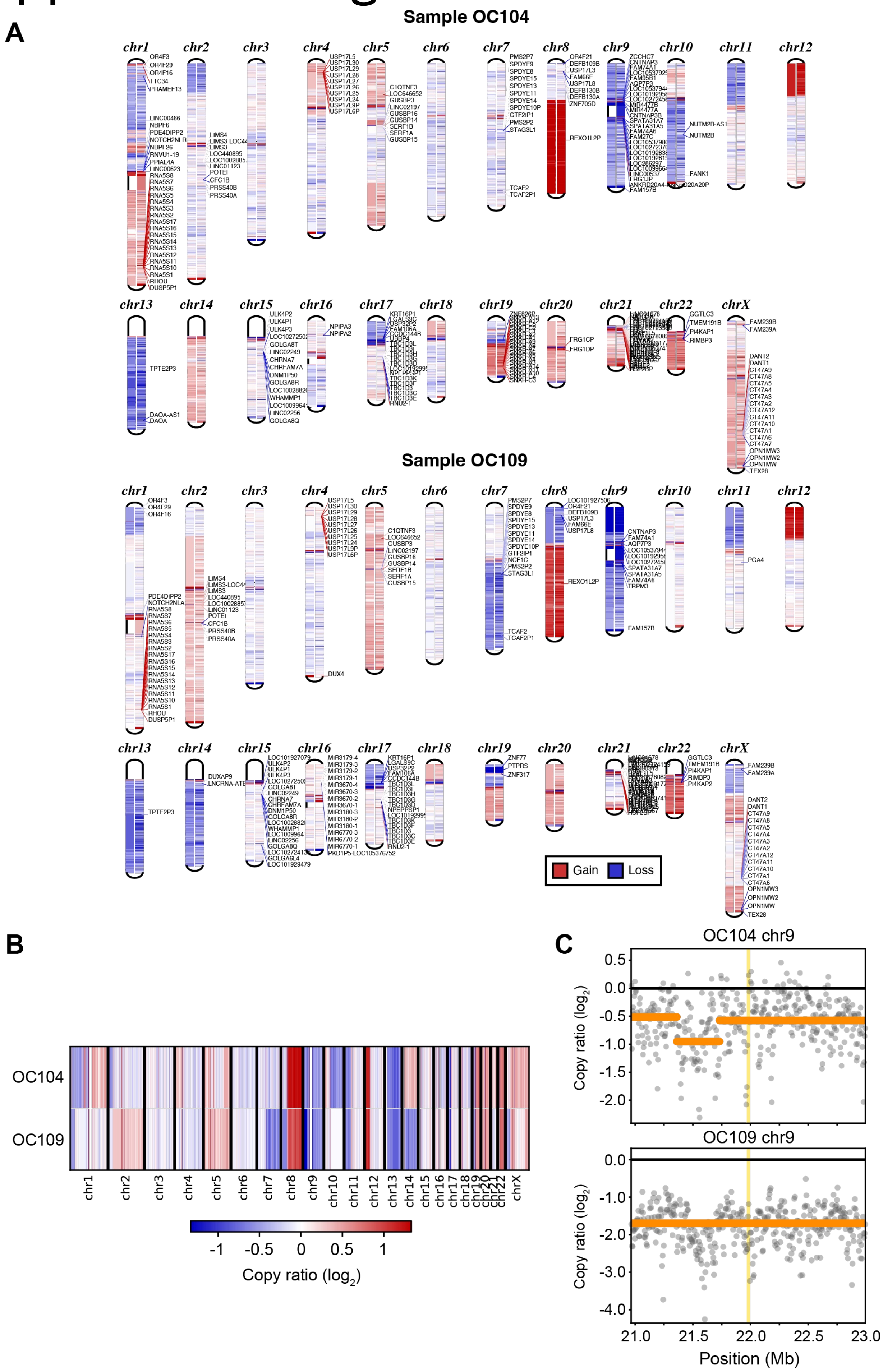

bioRxiv preprint doi: https://doi.org/10.64898/2026.07.06.736185; this version posted July 10, 2026. The copyright holder for this preprint (which was not certified by peer review) is the author/funder, who has granted bioRxiv a license to display the preprint in perpetuity. It is made available under a CC-BY 4.0 International license.

Supplemental Figure 4

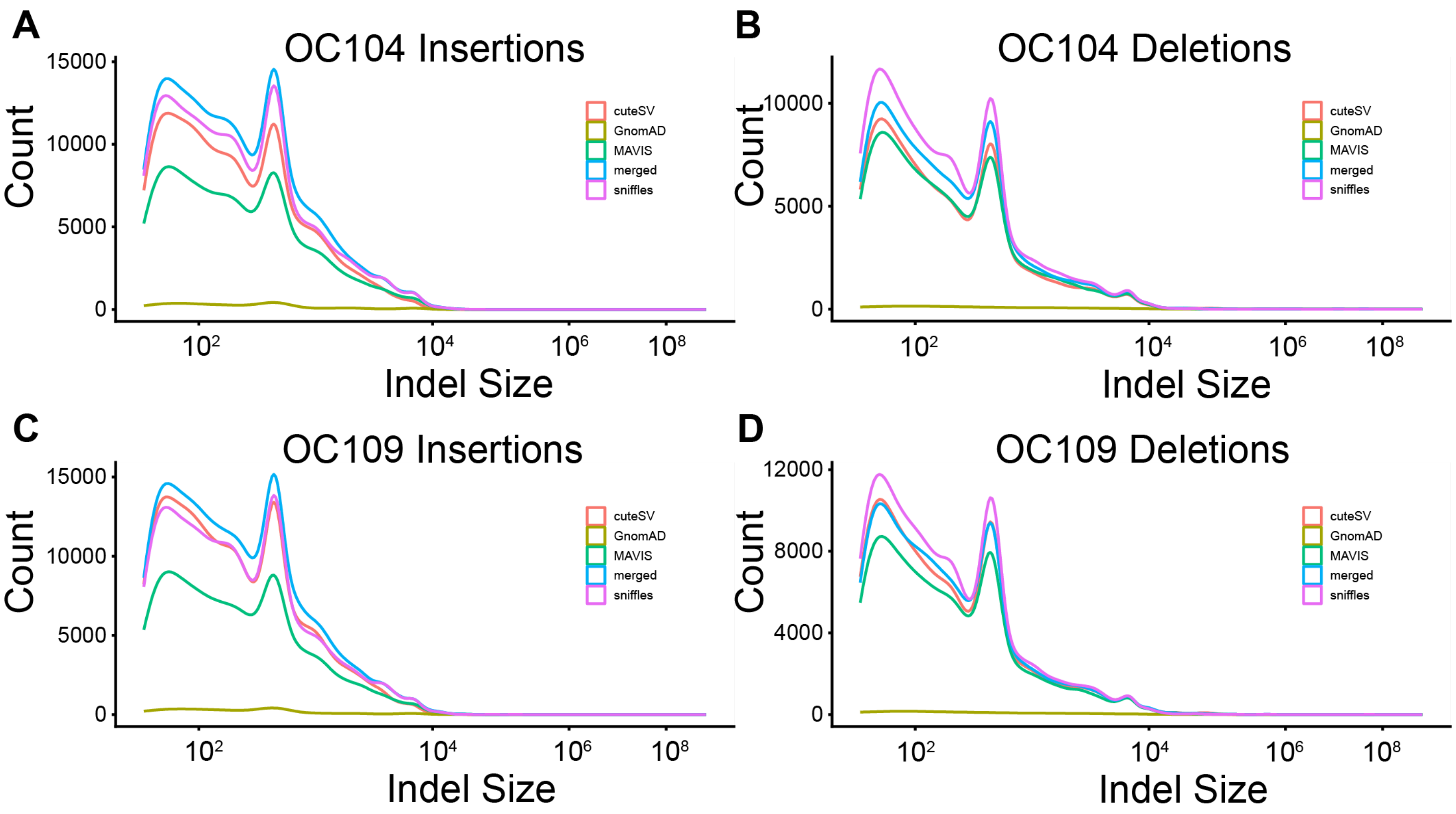

bioRxiv preprint doi: https://doi.org/10.64898/2026.07.06.736185; this version posted July 10, 2026. The copyright holder for this preprint (which was not certified by peer review) is the author/funder, who has granted bioRxiv a license to display the preprint in perpetuity. It is made available under a CC-BY 4.0 International license.

Supplemental Figure 5

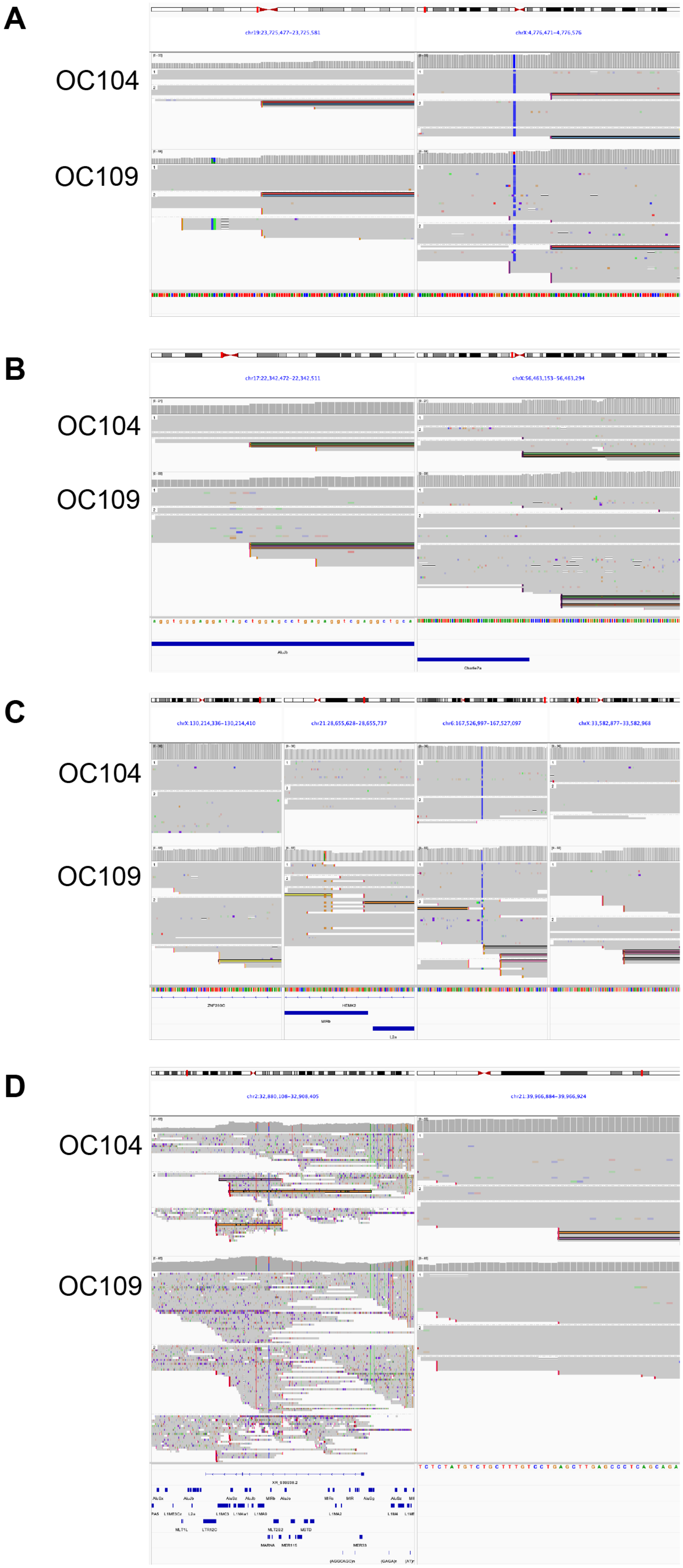

bioRxiv preprint doi: https://doi.org/10.64898/2026.07.06.736185; this version posted July 10, 2026. The copyright holder for this preprint (which was not certified by peer review) is the author/funder, who has granted bioRxiv a license to display the preprint in perpetuity. It is made available under a CC-BY 4.0 International license.

Supplemental Figure 6

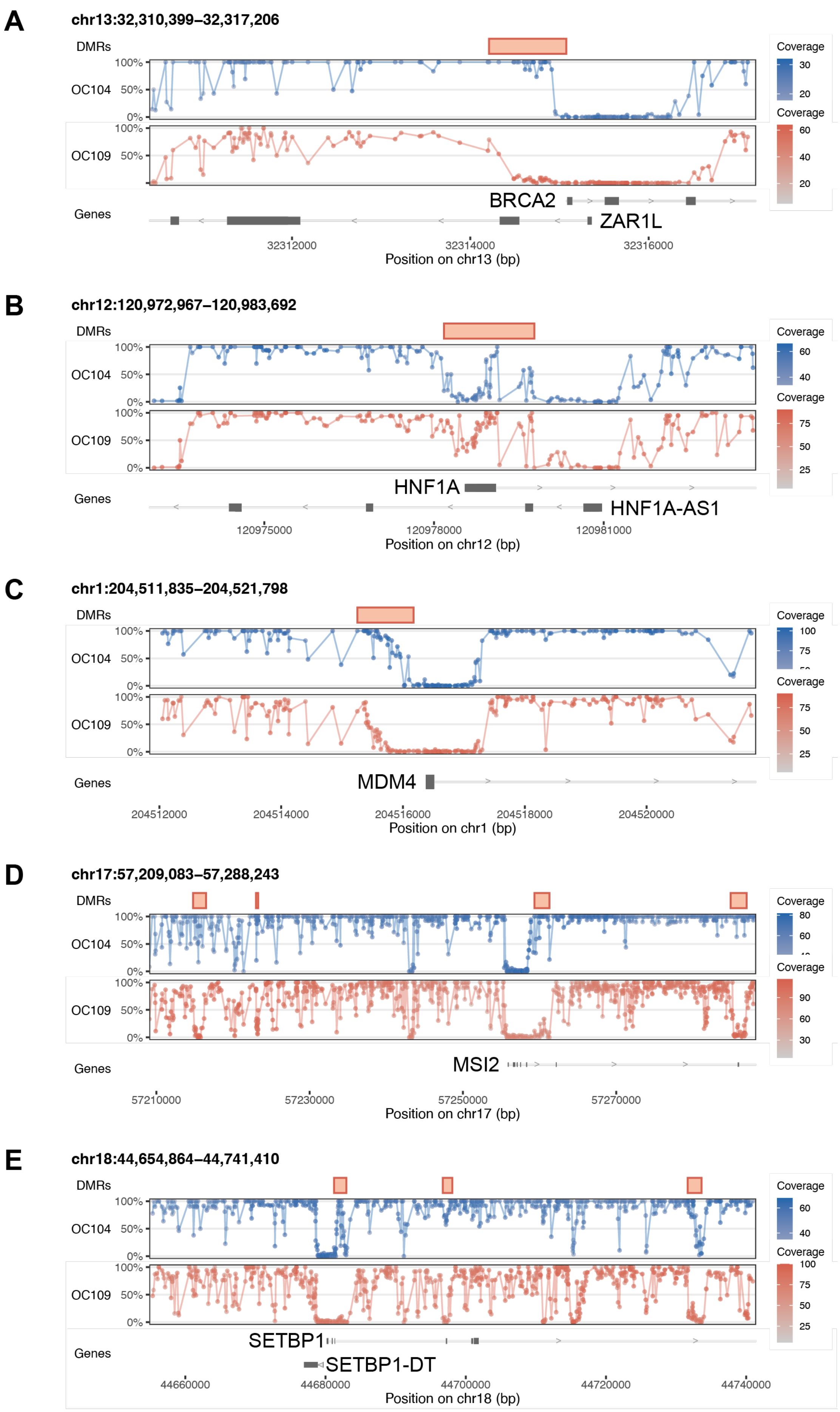

Supplement: Supplement 4 [file NIHPP2026.07.06.736185v1-supplement-4.pdf]
